# Injectable Hydrogels with Tissue-Adaptive Gelation and Mechanical Properties: Enhancing Softness and Stability

**DOI:** 10.3390/gels11120996

**Published:** 2025-12-11

**Authors:** Jessica Garcia, Foad Vashahi, Akmal Z. Umarov, Evgeniy V. Dubrovin, Apollinariya Yu. Konyakhina, Elena N. Subcheva, Dimitri A. Ivanov, Andrey V. Dobrynin, Sergei S. Sheiko

**Affiliations:** 1 Department of Chemistry, University of North Carolina at Chapel Hill, Chapel Hill, NC 27599, USA; jgarcia2@unc.edu (J.G.); foadvashahi@outlook.com (F.V.); 2 Faculty of Chemistry, Lomonosov Moscow State University (MSU), Leninskie Gory, 1-3, 119991 Moscow, Russia; umarovakmalum@gmail.com (A.Z.U.); dubrovin@polly.phys.msu.ru (E.V.D.); dimitri.ivanov@uha.fr (D.A.I.); 3 Scientific Center for Genetics and Life Sciences, Sirius University of Science and Technology, 1 Olympic Ave, 354340 Sochi, Russia; konyahina.ay@talantiuspeh.ru (A.Y.K.); subcheva.en@talantiuspeh.ru (E.N.S.); 4 Institut de Sciences des Matériaux de Mulhouse-IS2M, CNRS UMR 7361, 15, Rue Jean Starcky, 68057 Mulhouse, France

**Keywords:** bottlebrush polymers, thermosensitive hydrogels, tissue-mimetic mechanical properties, injectable tissue fillers

## Abstract

Ultra-soft injectable hydrogels are paramount in biomedical applications such as tissue fillers, drug depots, and tissue regeneration scaffolds. Synthetic approaches relying on linear polymers are confronted by the necessity for significant dilution of polymer solutions to reduce chain entanglements. Bottlebrush polymers offer an alternative approach due to suppressed chain overlap and entanglements, which facilitates lower solution viscosities and increased gel softness. Leveraging the bottlebrush architecture in linear-bottlebrush-linear (LBL) block copolymer systems, where L is a thermosensitive linear poly(N-isopropylacrylamide) block, and B is a hydrophilic polyethylene glycol brush block, injectable hydrogels were designed to mimic tissues as soft as the extracellular matrix at high polymer concentrations. Compared to an analogous system with shorter brush side chains, increasing the side chain length enables a decrease in modulus by up to two orders of magnitude within 1–100 Pa at 20 wt% polymer concentrations, near to the physiological water content of ~70%. This system further exhibits thermal hysteresis, enabling stability with inherent body temperature fluctuations. The observed features are ascribed to kinetically hindered network formation by bulky macromolecules.

## 1. Introduction

Finding a synthetic replacement with the mechanical characteristics of the extracellular matrix (ECM) is vital in the development of biomedical implants and tissue regeneration [1,2,3,4]. The ECM represents a hierarchical, ultra-soft collagen scaffold that holds controlled amounts of water (~70%), providing mechanical support and environment for cellular processes [5,6,7]. The ECM modulus varies within the range of 10–10^3^ Pa, depending on the type of tissue, and it is crucial for cellular functions, such as proliferation and migration [5,8,9]. Therefore, mimicking tissues’ mechanical properties reduces the risk for immune rejection of biomedical implants [10]. Polymer hydrogels have been extensively used to mimic the ECM mechanics [11,12,13,14,15,16]. Particular attention has been paid to in situ-forming injectable gels, which provide a minimally invasive approach and adopt unique shapes filling irregular cavities [17,18,19,20]. However, polymeric hydrogels based on linear polymer networks require exceedingly large amounts of water at low polymer concentrations to replicate tissue softness and low solution viscosities for injectability. This inherent limitation of linear polymer systems stems from the requirement for significant dilution (90–99% water) to achieve a tissue-mimicking modulus below 10^3^ Pa [21,22]. Exceedingly large amounts of water worsen their mechanical strength, leading to breakage under in-body stresses [23,24]. In addition, accessing a wide range of moduli at a constant water content to target different tissues is challenging in linear polymer gels, since the mechanical and swelling properties are coupled through crosslink density [22]. For example, low-solution viscosities result in more mechanically robust materials but require higher injection forces, which is difficult in fine needles for minimally invasive deliveries [25].

**Figure 1:**
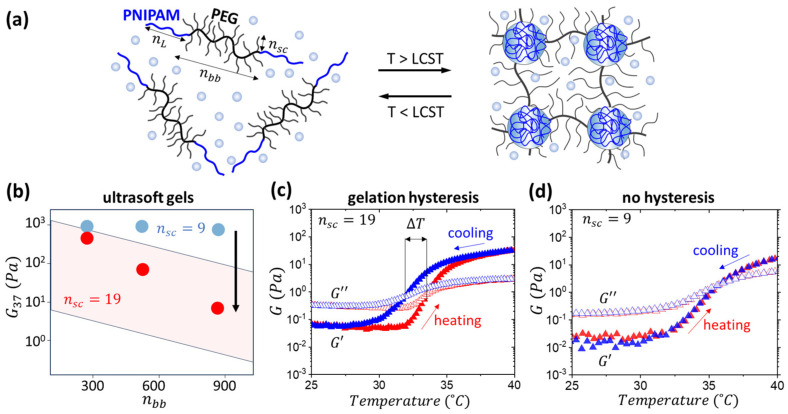
(**a**) Microphase separation of the linear PNIPAM and brush PEG block results in reversible self-assembly of a hydrogel network upon heating above the lower critical solution temperature (LCST) of PNIPAM, ranging from 30 to 40 °C. (**b**) Hydrogels with longer side chains demonstrated a significant decrease in modulus at 37 °C, spanning two orders of magnitude at a polymer concentration of 20 wt.%. The blue and red data points correspond to representative hydrogel samples with the same volume fraction of PNIPAM (ϕ_L_ = 0.2), but different DPs of PEG side chains n_sc_ = 9 and n_sc_ = 19, respectively. The pink parallelogram depicts the modulus spread of the n_sc_ = 19 samples synthesized in this study and discussed below. (**c**) Hydrogels with longer side chain (n_sc_ = 19) (n_bb_ = 580, ϕ_L_ = 0.12) exhibit thermal hysteresis shown by the differences in the heating and cooling cycles. (**d**) In contrast, hydrogels with shorter side chains (n_sc_ = 9) yet similar composition (n_bb_ = 550, ϕ_L_ = 0.12) do not show thermal hysteresis upon heating and cooling.

The above limitations can be resolved by implementing bottlebrush hydrogels, where the brush architecture allows for significantly lowering both solution viscosity and gel modulus by reducing chain entanglements. The densely grafted side chains act as cosolvent, reducing the need for large amounts of water, and the architectural diversity of bottlebrush networks allows for modulus variation at a given water content or even in a dry state [21,26,27,28,29,30]. Recently, the concept of injectable tissue-mimetic hydrogels has been implemented in linear-bottlebrush-linear (LBL) triblock copolymers with thermosensitive linear poly(N-isopropylacrylamide) (PNIPAM) and polyethylene glycol (PEG) bottlebrush (bb) blocks (Figure 1a) [31]. Controlled gelation at body temperature, yielding hydrogels with a modulus in the 1–10^3^ Pa range has been reported. However, the modulus below 10^2^ Pa was only achieved at a high water fraction of 90–95 wt%, which is significantly higher than the physiological water content of ca. 70% and may cause osmotic flow across the interface between an injected implant and the surrounding tissue. Herein, we leveraged the bottlebrush architecture to prepare hydrogels with an ECM-like modulus at lower water content. The goal of this study is to explore the effect of degrees of polymerization (DP) of side chains (nsc) and backbone (nbb) on gelation temperature and modulus of PNIPAM-bbPEG-PNIPAM hydrogels, at a given fraction of PNIPAM. The gelation process and corresponding rheological behavior were monitored by small-angle X-ray scattering (SAXS) and rheology to achieve a superlow modulus in the 1 to 100 Pa range at a 20 wt% polymer concentration (Figure 1b). Notably, the two-order of magnitude modulus variation was realized at constant water content by tuning the triblock structure only. Due to increased molecule size, this new system displayed the thermal hysteresis between gelation and gel–sol temperature (Figure 1c) otherwise not apparent in hydrogels with shorter side chains (Figure 1d). The temperature gap between the gelation and gel–sol temperatures provides additional stability against inherent body temperature variations (36.5–37.5 °C) (Figure 1d). Exploiting the brush architecture allows us to conform to a wide range of moduli mimicking the ECM, making LBL a suitable platform for various cell cultures and tissue regeneration. The biocompatibility of PNIPAM-bbPEG-PNIPAM hydrogels was validated by cytotoxicity tests and in vivo injection to animal models [31].

## 2. Results and Discussion

**Figure A1:**
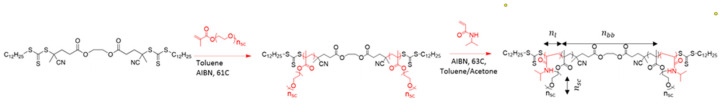
Synthetic scheme of the 2-step synthesis of PNIPAM-bbPEG-PNIPAM triblock copolymers using RAFT polymerization. In the first step the brush block is made via heat-initiated RAFT using PEG-MA (Mn 950) macromonomers. In the second step, NIPAM monomers are polymerized from the bbPEG to obtain the linear blocks.

**Figure A2:**
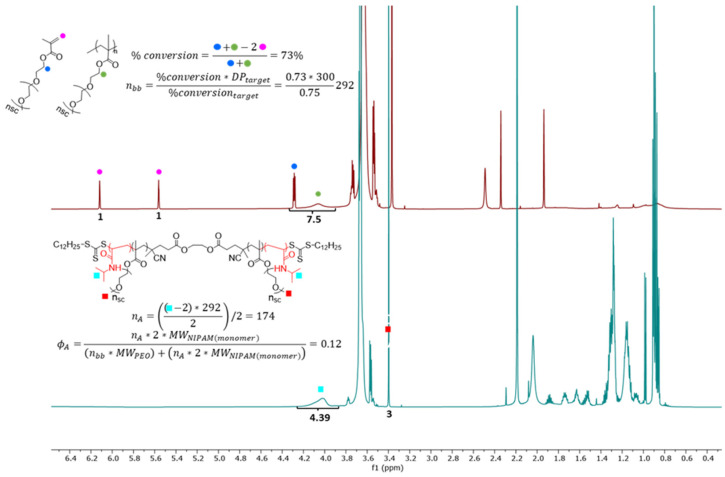
^1^H NMR (600 Hz, CDCl_3_) of final conversion for synthesizing bbPEG with DP = 292 and subsequent polymerization of the L-blocks with a final ϕ_L_ = 0.12. In the red spectrum, the conversion of polymerized PEG macromonomers via RAFT polymerization is calculated to obtain DP of backbone (n_bb_). In the blue spectrum, the volume fraction of PNIPAM is calculated relative to the PEG brush block.

To demonstrate the effect of side chain length on gelation and rheological behavior of bottlebrush hydrogels, PNIPAM-bbPEG-PNIPAM triblock copolymers with n_sc_ = 19 and different fractions of the PNIPAM block were synthesized in two steps (Figure A1 and Figure A2) as described elsewhere [31]. First, PEG bottlebrushes with three different backbone DPs (n_bb_ = 290, 580, 890) were synthesized by Reversible Addition Fragmentation Chain Transfer (RAFT) polymerization of poly(ethylene-glycol) methyl ether methacrylate (PEGMA) using a difunctional chain transfer agent (CTA). Subsequently, linear PNIPAM blocks were grown on both ends of the PEG bottlebrush block to systematically vary PNIPAM volume fraction (ϕL) between 5 and 20% (Table 1). For direct comparison with the previous study of the n_sc_ = 9 systems [31], solutions ranging from 5 to 20 wt% were prepared to monitor gelation using ultra-small and small-angle X-ray scattering (USAXS-SAXS) and rheological measurements.

### 2.1. Structure of Individual Macromolecules and Networks

**Figure 2:**
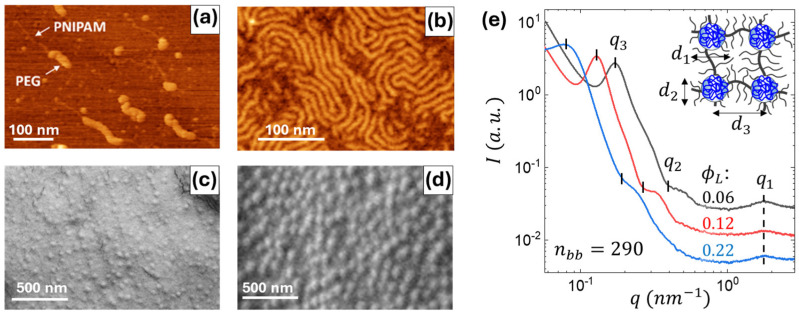
(**a**) AFM height image of individual brush-like triblock copolymer molecules (n_bb_ = 290, ϕ_L_ = 0.22) adsorbed on a modified graphite substrate under controlled shear flow. The image highlights the contrast between the thicker PEG bottlebrush blocks and the thinner PNIPAM linear blocks. (**b**) AFM height image of a densely packed monolayer of brush-like triblock copolymers (n_bb_ = 290, ϕ_L_ = 0.12) on a mica substrate. (**c**,**d**) SEM surface images of thick films reveal the evenly dispersed spherical PNIPAM domains for two samples: (**c**) n_bb_ = 580, ϕ_L_ = 0.07, (**d**) n_bb_ = 890, ϕ_L_ = 0.20. (**e**) SAXS curves of elastomers from the n_bb_ = 290 series with different volume fractions of the linear PNIPAM blocks (ϕ_L_) as indicated. The determined structural parameters (d_1_, d_2_, d_3_) are summarized in Table 1.

**Figure A3:**
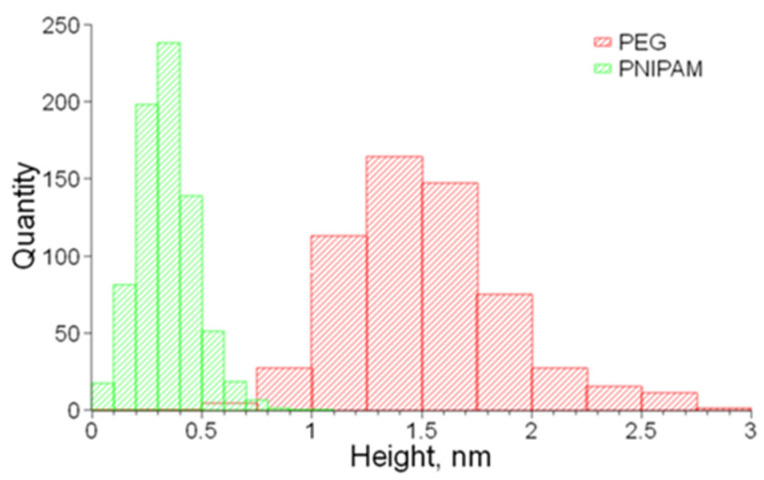
Height distribution histograms of bottlebrush PEG and linear PNIPAM blocks.

A series of structural studies were conducted to verify the triblock structure and microphase separation of the synthesized copolymers. Representative data are shown in Figure 2. Individual macromolecules were imaged by atomic force microscopy (AFM), where sparse and dense monolayers were deposited on graphite and mica and substrates, respectively. Special ultrasharp tips were used to obtain a clear resolution of both brush and linear blocks. For the imaging of the PNIPAM blocks, a controlled shear flow was applied during spin-coating of sparse monolayers to extend the linear chains (Figure 2a). The suggested designation of the constituting blocks (arrows) is based on the height analysis distributions, as shown in Figure A3, where thicker chains with a height of 1.5 ± 0.4 nm were attributed to PEG brush blocks, and thinner chains with a height of 0.34 ± 0.13 nm were attributed to PNIPAM linear blocks. In both dispersed (Figure 2a) and densely packed (Figure 2b) states, the brush blocks appeared as wormlike species, corroborating previous observations of bottlebrush macromolecules [31]. In thick films, scanning electron microscopy (SEM) images demonstrate well-defined spherical domains (Figure 2c,d), suggesting microphase separation of the PNIPAM and PEG blocks, corroborated by SAXS (Figure 2e). The domain size (d_2_) and interdomain distance (d_3_) increase with the degree of polymerization of the PNIPAM block (Table 1), which is consistent with the previous studies of LBL triblock copolymers [27,31]. The smaller molecules with n_bb_ = 290 follow the theoretical prediction for the aggregation number (Q) increasing with n_L_. However, larger molecules demonstrate an inconsistent behavior, i.e., Q decreasing with n_L_, ascribed to kinetic hindrance, which is also manifested in modulus decreasing with n_bb_ as discussed below. The very large Q = 2166 and 3134 are due to transitioning to cylindrical morphology at high PNIPAM volume fractions [27].

**Figure 3:**
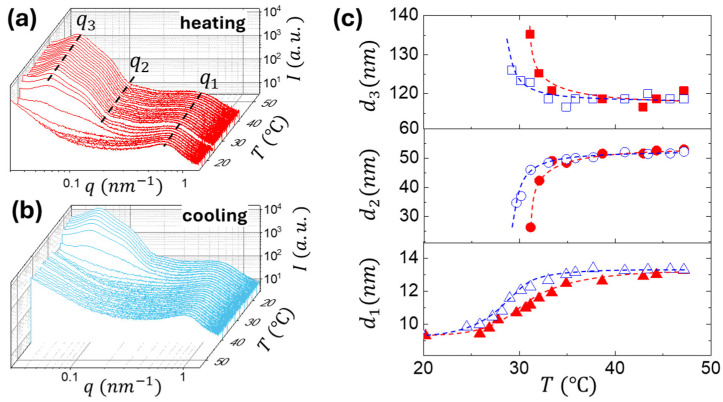
Temperature-dependent USAXS-SAXS profiles of PNIPAM–bbPEG–PNIPAM bottlebrush copolymer (n_bb_ = 580 and ϕ_L_ = 0.20 at 15 wt%) recorded during (**a**) heating (15–50 °C) and (**b**) cooling (50–15 °C). (**c**) Evolution of structural parameters during the heating-cooling cycle: inter-brush spacing (d_1_ = 2π/q_1_), PNIPAM domain size (d_2_), determined by fitting the experimental scattering curves using a spherical form factor P(q), convoluted with a Gaussian size distribution at q_2_, and inter-domain distance (d_3_ = 2π/q_3_).

In situ USAXS-SAXS gelation experiments were performed by continuously heating and cooling the polymer solutions in water from 15 to 20 °C at 1.0 K/min (Figure 3a,b). During heating above LCST, the appearance of form-factor oscillations ($q_{2}$) along with the structure-factor peak ($q_{3}$) signals the formation of spherical domain due to microphase separation of the temperature sensitive PNIPAM blocks. The temperature dependence of the structural parameters (Figure 3c) reveals that the PNIPAM domains emerge at T > LCST and then grow in size (d_2_) upon heating, while the interdomain distance (d_3_) decreases due to the fusion of smaller domains. Both parameters level off at T > 35 °C. The $q_{1}$ peak, corresponding to the average interbrush distance $d_{1}=2\pi /q_{1}$, shifts toward the low-q region due to the water uptake by the bottlebrush phase upon the collapsing of the PNIPAM domains. The system exhibits full reversibility upon cooling: both the structural and form-factor features associated with the PNIPAM domains vanish at T < LCST, returning the system to the solution state. Note that the d_1_, d_2_, and d_3_ temperature variations display the gelation hysteresis in agreement with the temperature sweeps of the dynamic moduli discussed below.

### 2.2. Monitoring the Gelation Process by Rheology

**Figure 4:**
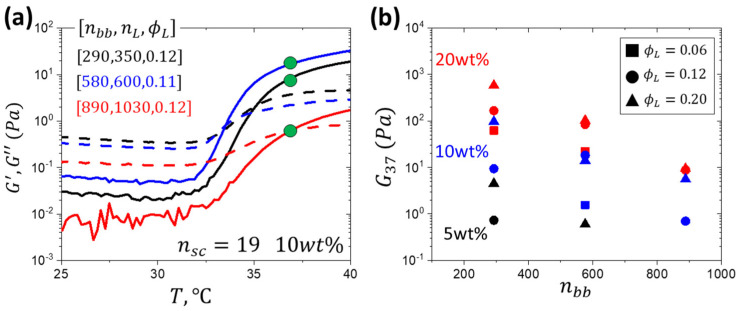
(**a**) Gelation curves of samples with similar volume fractions of PNIPAM but different lengths of the PNIPAM linear block (n_L_) and PEG brush block (n_bb_) as indicated where the green dots indicate the storage modulus at 37 °C. (**b**) Storage modulus at 37 °C at different solution concentrations (5, 10, 20 wt%) and volume fractions of PNIPAM blocks (ϕ_L_) covers a wide range of moduli (1 to 10^3^ Pa). At given ϕ_L_ and n_bb_, the modulus decreases with decreasing solution concentration.

**Figure A4:**
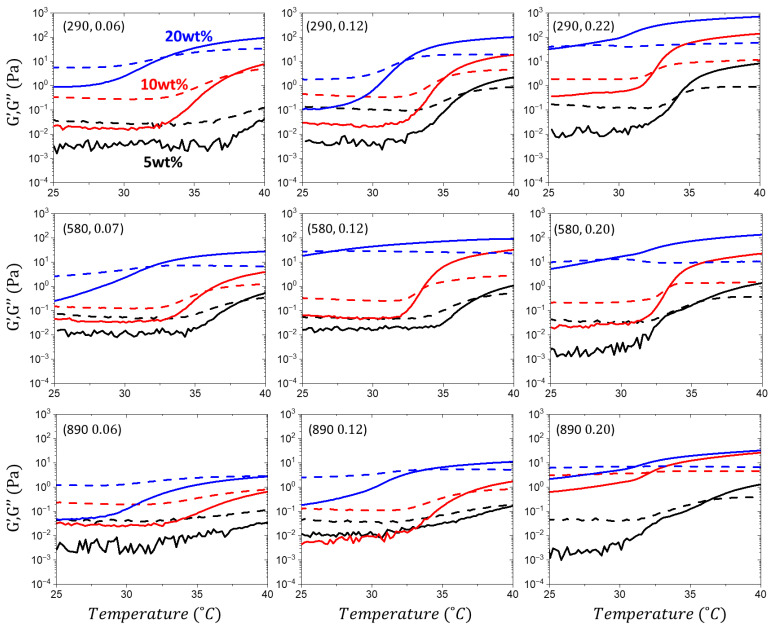
Heating cycle and gelation. Temperature ramps from 25 to 40 °C at a frequency of 1 Hz to monitor storage (G′) and loss (G″) moduli of PNIPAM-bbPEG-PNIPAM aqueous solution at three different polymer concentrations (5, 10, and 20 wt.%), as indicated. The G′ and G″ curves are identified as solid and dashed lines, respectively. The triblock compositions are indicated in parentheses as (n_BB_, ϕ_L_).

**Figure A5:**
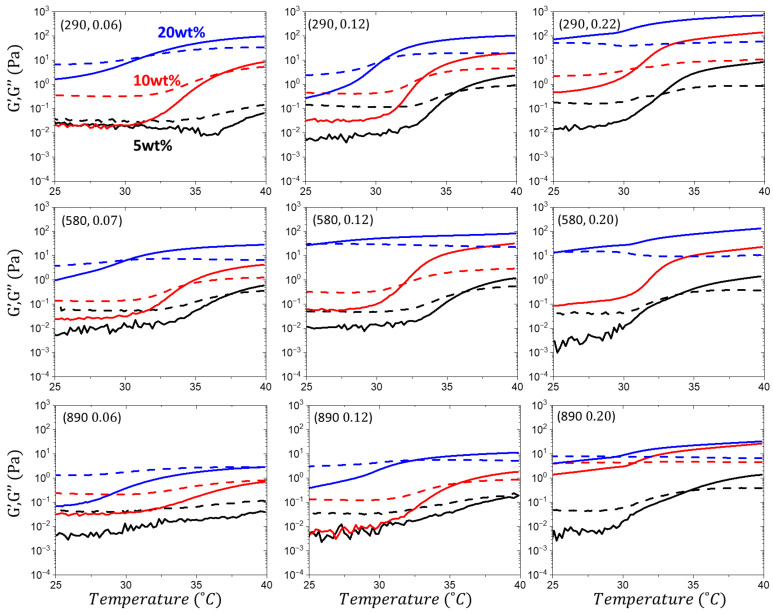
Cooling cycle and reversibility. Temperature ramps from 40 to 25 °C at a frequency of 1 Hz to monitor the storage (G′) and loss (G″) moduli to monitor the transition to a liquid state upon cooling. The transition temperature is identified by the crossover between the G′ and G″ curves. Curve legends and experimental conditions are consistent with Figure A4.

Variation in storage (G′) and loss (G″) shear moduli with temperature was monitored at different solution concentrations (Figure A4 and Figure A5). Specifically, we were interested in the effect of the triblock architecture on the gel modulus at 37 °C (G_37_), depicted by green dots in Figure 4a. By varying solution concentration, a wide range of modulus (1–10^3^ Pa) was covered, effectively mapping the softness of the ECM at high polymer concentrations (Figure 4b, Table A1). Notably, at 20 wt%, a modulus as low at 10 Pa was achieved, otherwise only achievable at 5 wt% solution concentration with n_sc_ = 9 hydrogels [31]. Increasing side chain length dilutes crosslink density, yielding looser networks as seen by the increase in the d_3_ distance in comparison to n_sc_ = 9. At a given solution concentration and triblock composition, e.g., ϕ_L_ = 0.2, the modulus of the n_sc_ = 19 systems decreases with increasing nbb by two orders of magnitude. This behavior significantly differs from the systems with shorter side chains (n_sc_ = 9), which maintain a nearly constant modulus across various n_bb_ values (Figure 1b). The observed decrease in modulus is ascribed to kinetically hindered association of much larger LBL macromolecules with longer side chains, supported by the decrease in aggregation numbers with n_L_ for the n_bb_ = 580 and 890 series. The combined effect of slower mobility of larger macromolecules and the higher barrier for PNIPAM association due to longer PEG side chains may lead to lower crosslink density and dangling strands.

**Figure 5:**
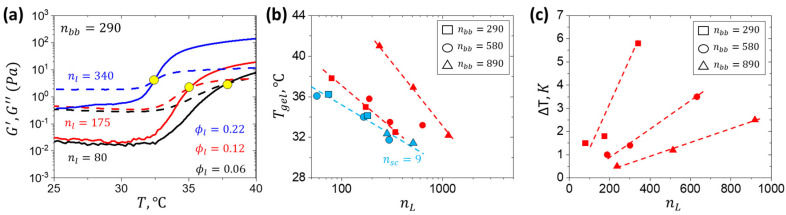
(**a**) Gelation curves of samples with different DPs of the linear PNIPAM block at 10 wt% polymer concentration indicate T_gel_ by the crossover of the storage (G′) and loss (G″) modulus curves marked by the yellow dot. (**b**) Gelation temperature measured at the G′ and G″ crossover point using shear rheology in a cone and plate geometry at 10 wt% as a function of n_L_. (**c**) Gelation–solvation hysteresis measured as temperature difference (ΔT) at the G′ and G″ crossover between heating and cooling cycles at 10 wt%.

Gelation temperature (T_gel_) is identified as a crossover of the G′ and G″ curves (Table A1) [32,33,34]. Consistent with the previously reported n_sc_ = 9 hydrogels [31], T_gel_ increases with decreasing polymer concentration and PNIPAM volume fraction ϕ_L_ (Figure 5a). However, hydrogels with n_sc_ = 19 demonstrate a strong dependence of T_gel_ on n_bb_ (Figure 5b), indicated by a distinct slope for each data set at different n_bb_ values in contrast to a collapse into the same trendline for the system with shorter side chains. In addition, T_gel_ spanned a range of approximately 10 °C at constant n_L_. Consistent with the SAXS data (Figure 3c), hydrogels with longer side chains exhibited notable thermal hysteresis, as evidenced by heating–cooling transition differences ranging from 0.5 to 6 °C (Figure 5c). The hysteresis is attributed to larger domains of entangled linear PNIPAM chains, which hinders network disassembly.

## 3. Conclusions

The bottlebrush architecture provides a versatile structural platform for the design of injectable hydrogels with tissue-mimetic softness. In PNIPAM-bbPEG-PNIPAM linear-bottlebrush-linear triblock copolymers, the brush architectural parameters were independently tunned, enabling an advanced system to achieve a wide range of moduli mimicking biological tissues at controlled water fractions. The reversible nature of this thermosensitive system, as demonstrated by SAXS and rheological analysis, enables minimally invasive injections at physiologically relevant temperatures. Notably, increasing the side chain length demonstrated a strong dependence of modulus and gelation temperature on n_bb_, unlocking a mechanism to attain ECM-like softness at high polymer concentrations. Additionally, the system exhibited thermal hysteresis, enhancing its stability between heating and cooling cycles.

## 4. Materials and Methods

*Materials*: Toluene, hexanes, chloroform, azobisisobutyronitrile (AIBN), and activated basic alumina were purchased from Sigma Aldrich, St. Louis, MO, USA; magnesium sulfate (MgSO_4_) was purchased from Thermo Fisher Scientific, Waltham, MA, USA and used as received. Poly(ethylene glycol) methyl ether methacrylate (PEG-MA) was purchased from Sigma Aldrich and purified with activated basic alumina to remove inhibitors before polymerization. N-isopropylacrylamide was purchased from TCI and recrystallized using a 50:50 toluene/hexane mixture (three times). PNIPAM-PEG-PNIPAM triblock copolymer were synthesized as previously reported. 

*Rheological Data*: Samples were prepared at 3, 10, 20% in water for all synthesized materials. All measurements were made using the Discovery Series HR30 rheometer from TA Instruments, New Castle, DE, USA, equipped with a solvent trap to prevent evaporation. Stainless-steel cone-plate (40 mm and 0.04 rad) geometry was used to monitor gelation ranging from 20 to 40 °C with a rate of 1 °C/min at a frequency of 1 Hz.

*Scanning Electron Microscopy (SEM)*: The phase-separated surface morphology was examined using a Carl Zeiss CrossBeam 550 SEM (Carl Zeiss Microscopy GmbH, Jena, Germany) operated at an accelerating voltage of 80 kV.

*Ultra-small and Small-Angle X-ray Scattering (USAXS–SAXS)*: Experiments were conducted at beamline ID02 of the European Synchrotron Radiation Facility (ESRF, Grenoble, France) in transmission geometry, using 12 keV photon energy. The accessible scattering vector range was |q| = 4π sin(θ)/λ, spanning from 2.0 × 10^−2^ to 1.5 nm^−1^. Scattered intensity was recorded with an Eiger2 4M detector, DECTRIS Ltd., Baden-Dättwil, Switzerland (75 μm pixel size) positioned 5.0 m from the sample. Temperature was controlled between 15 °C and 55 °C using a custom-built capillary stage with a Peltier element. Data correction, calibration, and integration were performed using a fast azimuthal integration Python library (version pyFAI 2025.3.0).

*Atomic Force Microscopy (AFM)*: AFM imaging of individual macromolecules was performed by depositing 5 μL of a 0.01 g/L polymer solution onto plasma-treated, freshly cleaved mica. The droplet was immediately removed with an air stream to achieve uniform dispersion. Imaging was carried out using a Bruker JPK NanoWizard Ultraspeed 2, Bruker, Berlin, Germany in intermittent contact mode (air, 1 Hz scan rate), equipped with RTESPA-300 cantilevers, Bruker, Tucson, AZ, USA (resonance frequency 200–400 kHz, stiffness 40 N/m).

For AFM imaging of the PNIPAM end-capping blocks, a 2 μL droplet of 0.01 g/L PEG–PNIPAM 292-24 solution in 0.5 M KCl was spin-coated onto GM-functionalized graphite. 2 Measurements were performed on an Ntegra Prima AFM (NT-MDT, Moscow, Russia) using ultrasharp tips (carbon nanowhiskers grown on commercial silicon cantilevers, spring constant 5–30 N/m) operated in attractive-mode intermittent contact.

## Figures and Tables

**Table 1:** Architectural and morphological parameters of PNIPAM-bbPEG-PNIPAM elastomers.

n_sc_ ^1^	n_bb_ ^2^	n_L_ ^3^	ϕ_L_ ^4^	d_1_ ^5^	d_2_ ^6^	d_3_ ^7^	Q ^8^
19	290	80	0.06	3.6	23.2	37.9	474
		175	0.12		34.9	51.9	748
		340	0.22		45.3	84.6	932
19	580	190	0.07	3.6	35.8	59.6	739
		300	0.12		36.1	60.5	480
		630	0.20		76.4	74.9	2166
19	890	240	0.06	3.6	29.4	62.8	328
		510	0.12		37.6	67	316
		920	0.20		98.0	132.6	3134

Degrees of polymerization of ^1^ PEG side chains, ^2^ bottlebrush backbone, and ^3^ PNIPAM linear blocks. ^4^ Volume fraction of PNIPAM linear blocks. The microphase separated structure of the synthesized PNIPAM-bbPEG-PNIPAM triblock copolymers in a dry state is described by ^5^ interbrush distance determined as $d_{1}=2\pi /q_{1}$, where $q_{1}$ corresponding to the backbone-backbone correlation, ^6^ diameter of the spherically shaped PNIPAM domains, determined by fitting with the form factor of spheres ($q_{2}$), and ^7^ distance between the PNIPAM domains ($d_{3}=2\pi /q_{3}$). ^8^ Aggregation number determined as $Q=\pi d_{2}^{3}/\left(6n_{L}v_{L}\right)$, where $v_{L}=M_{0}/\left(\rho N_{Av}\right)=0.171\;{nm}^{3}$ is volume of NIPAM monomer.

## Data Availability

The raw data supporting the conclusions of this article will be made available by the authors on request.

## Abbreviations

The following abbreviations are used in this manuscript:

LBL	Linear-bottlebrush-linear
Bb	Bottlebrush
ECM	Extracellular matrix
PNIPAM	Poly(N-isopropylacrylamide)
PEG	Polyethylene glycol
PEGMA	Poly(ethylene-glycol) methyl ether methacrylate
CTA	Chain transfer agent
RAFT	Reversible addition fragmentation chain transfer
LCST	Lower critical solution temperature
USAXS-SAXS	Ultra-small and small-angle X-ray scattering
DP	Degree of polymerization
AFM	Atomic force microscopy
SEM	Scanning electron microscopy

## Appendix A

### Appendix A.1. Synthesis and Molecular Characterization

Polyethylene Glycol brush (bbPEG) synthesis. PEG-MA (Sigma-Aldrich, St. Louis, MO, USA, Mn 950), bifunctional CTA, and AIBN initiator were dissolved in toluene in a Schlenk flask equipped with a stir bar. The reaction mixture was sparged with nitrogen for 1 h. Polymerization began upon placing the reaction flask at 61 °C. Reaction conversion was tracked by ^1^H NMR targeting 75% conversion and stopped by exposing the reaction to air. The polymer was purified by precipitating from toluene/hexanes in triplicate).

PNIPAM-bbPEG-PNIPAM synthesis. In a Schlenk flask, bbPEG and AIBN initiator were dissolved in a solution of toluene/acetone (3:1). The solution was sparged with nitrogen for 1 h. The reaction flask was placed at 63 °C to start polymerization. After 3 h, polymerization was stopped by exposing the reaction to air. The polymer was purified by precipitating in acetone/hexanes in triplicate. The purified polymer was dried by casting in a Teflon Petri dish, the volume fraction of the PNIPAM blocks was determined by ^1^H NMR.

### Appendix A.2. Rheology Data

**Table A1:** Rheological data for hydrogels of n_sc_ = 19 at different architectural compositions and solution concentrations.

$n_{bb}$ ^(1)^	$n_{L}$ ^(2)^	$\phi_{L}$ ^(3)^	wt. % ^(4)^	$T_{gel}\left({}^{\circ}C\right)-Heat$ ^(5)^	$G_{gel}\left(Pa\right)-Heat$ ^(5)^	$T_{gel}\left({}^{\circ}C\right)-Cool$ ^(5)^	$G_{gel}\left(Pa\right)-Cool$ ^(5)^	$G_{37}\left(Pa\right)$ ^(6)^	Hysteresis (K) ^(7)^
290	80	0.06	5	N/A	N/A	N/A	N/A	0.00428	N/A
			10	37.9	3.08	37.0	3.10	1.96	0.9
			20	33.0	15.5	31.5	14.8	62.2	1.5
	174	0.12	5	36.6	0.51	35.7	0.51	0.73	0.9
			10	35.1	2.13	33.3	1.9	9.43	1.8
			20	32.3	11.0	30.5	10.0	77.5	1.8
	341	0.22	5	34.2	0.385	32.6	0.380	4.49	1.6
			10	32.5	3.90	30.9	4.75	96.1	1.6
			20	26.8	44.5	21.0	46.2	580	5.8
580	188	0.07	5	38.4	0.259	37.5	0.260	0.177	0.9
			10	35.9	0.673	34.3	0.622	1.57	1.6
			20	31.8	6.58	30.1	6.64	22.4	1.7
	301	0.12	5	37.4	0.335	36.3	0.320	0.278	1.1
			10	33.5	0.812	31.9	0.677	18.34	1.6
			20	27.3	29.2	25.8	0.684	84.1	1.5
	632	0.20	5	35.5	0.232	33.9	0.257	0.343	1.6
			10	33.4	0.912	31.6	0.823	14.0	1.8
			20	28.8	12.9	25.3	14.2	102	3.5
890	237	0.06	5	N/A	N/A	N/A	N/A	0.0119	N/A
			10	N/A	N/A	N/A	N/A	0.261	N/A
			20	N/A	N/A	N/A	N/A	1.95	N/A
	512	0.12	5	N/A	N/A	N/A	N/A	0.0637	N/A
			10	36.9	0.621	35.7	0.632	0.650	1.2
			20	33.9	5.34	32.5	5.53	8.83	1.4
	919	0.20	5	36.3	0.298	34.7	0.303	0.470	1.6
			10	33.2	4.12	30.7	4.20	18.1	2.5
			20	31.1	7.04	29.4	7.62	24.8	1.7

Degrees of polymerization of ^(1)^ bottlebrush backbone and ^(2)^ PNIPAM linear blocks. ^(3)^ Volume fraction of PNIPAM linear blocks. ^(4)^ Concentration of polymer solution in water. T_gel-Heating_ and T_gel-cooling_ measured by the G′ G″ crossover corresponding to the heating cycle (Figure A4) and cooling cycle (Figure A5). ^(5)^ Temperature and G′ values corresponding to the gelation and gel–sol transitions measured during heating and cooling cycles, respectively (Figure A4 and Figure A5). ^(6)^ G_37_ is the storage modulus at 37 °C measured during the heating cycle (Figure A4). ^(7)^ Difference between the gelation and gel–sol transition temperatures.
